# Systematic LC/MS/MS Investigations for the IND-Enabling Extended Characterization of Antibody–Drug Conjugate Modifications

**DOI:** 10.3390/antib7040040

**Published:** 2018-11-16

**Authors:** Thomas Linz, Dominick Yeo, Qiuting Hong, Wesley Zmolek, Jesse McFarland, Robyn M. Barfield, William E. Haskins, David Rabuka

**Affiliations:** Catalent Biologics, 2600 Hilltop Drive, Richmond, CA 94806, USA; thomas.linz@catalent.com (T.L.); dominick.yeo@catalent.com (D.Y.); qiuting.hong@catalent.com (Q.H.); wesley.zmolek@catalent.com (W.Z.); jesse.mcfarland@catalent.com (J.M.); robyn.barfield@catalent.com (R.M.B.)

**Keywords:** antibody–drug conjugate, ADC, biologic, analytical characterization, SMARTag^®^

## Abstract

We hypothesized that systematic liquid chromatography-tandem mass spectrometry investigations of an antibody–drug conjugate (ADC), its small and large molecular components, and surrogate small-molecule conjugates might comprise a simple and efficient approach for the extended characterization of ADCs. Furthermore, we envisioned that results from this work might allow us to assign specific composition changes in the ADC based on monoisotopic mass shifts of conjugatable modifications as detected in the surrogate small-molecule conjugates. We tested our hypothesis with a case study using an aldehyde-tag-based ADC conjugated to a noncleavable linker bearing a maytansine payload. Nearly quantitative bioconversion from cysteine to formylglycine was observed in the monoclonal antibody, and bioorthogonal conjugation was detected only on the formylglycine residues in the ADC. Using our method, both conjugatable and nonconjugatable modifications were discovered in the linker/payload; however, only conjugatable modifications were observed on the ADC. Based on these results, we anticipate that our approach to systematic mass spectrometric investigations can be successfully applied to other ADCs and therapeutic bioconjugates for investigational new drug (IND)-enabling extended characterization.

## 1. Introduction

Therapeutic bioconjugates, including antibody–drug conjugates (ADCs), are an important class of biologic drugs that are well-represented in clinical trials for oncology and other indications. These leading-edge medicines, which harbor both chemical and post-translational modifications (PTMs) that require characterization, provide strong challenges for platform analytical methods that were built to address more traditional, less-complicated molecular formats, such as monoclonal antibodies. Moreover, the investigational new drug (IND)-enabling extended characterization of ADCs requires a full understanding of the small molecule (e.g., linker/payload) and large molecule (e.g., antibody and ADC) components to ensure consistent manufacturing, product and process quality, and safety and efficacy for patients [1,2].

Liquid chromatography-tandem mass spectrometry (LC/MS/MS) has been proposed as a multi-attribute method for IND-enabling extended characterization, quality control testing, and absorption, distribution, metabolism, and excretion (ADME) studies [3]. The latest high-resolution, exact-mass, ultrahigh-performance high-pressure LC/MS/MS instruments, methods, and algorithms can be leveraged to discover and quantify chemical modifications of small molecules (e.g., oxidation of linker/payloads), as well as assign chemical and post-translational modifications to specific amino acid residue(s) of large molecules (e.g., oxidation of methionine residues in antibodies and ADCs). LC/MS/MS provides monoisotopic mass (m) shifts and assignments to specific composition changes for small and large molecule components by measuring features in the mass-to-charge (*m*/*z*) and chromatographic dimensions. Modifications are identified by characteristic shifts in *m*/*z* for a given *z* and/or shifts in chromatographic retention times relative to the unmodified forms. Features are quantified by integrating the chromatographic peaks in extracted ion chromatograms (XICs) with a narrow *m*/*z* tolerance (e.g., 5 ppm) for each ion to characterize the relative abundance of various modifications in reference, partially degraded, and enriched minor variant samples.

The state-of-the-art analytic methods for ADCs—including LC/MS/MS techniques and orthogonal methods, such as hydrophobic interaction chromatography (HIC) to determine the average drug-to-antibody ratio (DAR)—have been described in a number of excellent reviews [1,4,5]. However, a typical LC/MS/MS approach to ADC analysis studies either the chemical modifications of small molecule components (e.g., linker/payloads and surrogate small-molecule conjugates) or the PTMs found on peptides released from digests of the large molecule components (e.g., unconjugated antibodies and ADCs). The results from these studies are typically correlated to intact mass results (LC/MS) for the large molecule components, particularly the average mass and average DAR observed for reduced, partially digested, or intact (either native [6] or denatured) ADCs. Thus, most ADC publications do not assess both the small molecule payload and peptide digest aspects of unconjugated antibodies and ADCs.

The IND-enabling extended characterization of ADCs requires direct correlation of results from both the small molecule and antibody aspects of LC/MS/MS studies, in addition to indirect correlations through the intact mass results and other supporting data (e.g., HIC) in order to gain a holistic understanding of modifications present in the ADC and its components. One of the areas of interest at the intersection of these datasets relates to the conjugatable modifications as compared to the nonconjugatable modifications observed in the linker/payload. These include making comparisons between modifications detected in the unconjugated or surrogate-conjugated small molecule payload and on the payload-modified peptides released from ADC digests. The identification of any unexpected conjugatable products would be particularly important. By studying the small molecule linker/payload in parallel with antibody and ADC digests, we also wanted to determine whether the observed modifications in each analyte were internally consistent across samples, thus lending greater support to the overall observations and conclusions.

To address these and other key questions systematically, we hypothesized that directed LC/MS/MS investigations of an ADC, its small and large molecular components, and surrogate small-molecule conjugates might be a simple and efficient approach for the extended characterization of ADCs. We tested our hypothesis with a case study on an ADC made with our proprietary SMARTag^®^ technology (Catalent Biologics, 2600 Hilltop Drive, Richmond, CA, USA) that uses an aldehyde-tagged antibody conjugated to a noncleavable linker bearing a maytansine payload [7]. 

## 2. Materials and Methods

### 2.1. Materials

The chemicals for this work were sourced from Sigma-Aldrich (St. Louis, MO, USA), with the exception of the Redwood Bioscience Inc. (RED)-106 linker/payload, the unconjugated antibody, and the RED-106-conjugated ADC. These were produced in-house using previously published methods [7,8,9].

### 2.2. Sample Preparation

Partially degraded RED-106 linker/payload was prepared by partial degradation of RED-106, where 0.1 M RED-106 was incubated in dimethylacetamide (DMA) at room temperature overnight.

RED-106(+IBA) and RED-106(+HMBA) conjugates were prepared by incubating RED-106 with isobutyraldehyde (IBA) or 3-hydroxy-4-methoxybenzaldehyde (HMBA) at 1:10 equivalent in 20 mM sodium citrate and 50 mM sodium chloride at pH 5.5 for 1 h or 28 h at 37 °C. The conjugation reaction was quenched with 10 mM butylated hydroxytoluene (BHT).

#### 2.2.1. In Vitro Proteolysis

Peptides from digests of unconjugated antibody or ADC were generated with trypsin. Reduction was performed with 20 mM dithiothreitol (DTT), and carbamidomethylation of cysteine residues (CAM) was performed with 50 mM iodoacetamide (IAA). At the end of proteolysis, the aldehyde group of fGly was labeled using 100 mM methoxylamine (MeOx) in 150 mM sodium citrate buffer at pH 5.5 in a 37 °C water bath overnight.

#### 2.2.2. LC/MS/MS Data Collection

A Q Exactive Hybrid Quadrupole-Orbitrap (QE) system (Thermo, San Jose, CA, USA) coupled with a binary ultrahigh performance high pressure LC pump (Agilent, Santa Clara, CA, USA), a linear reverse-phase 0.1% formic acid/acetonitrile gradient at 0.5 mL/min, and a C18 column (Zorbax, Eclipse plus C18, 2.1 × 100 mm, 1.8 μm, Agilent, Santa Clara, CA, USA) was employed. Data-dependent acquisition was performed where the top five most abundant ions were selected with a unique MS/MS scan function with 70 k mass resolution in the MS dimension and 17.5 k in the MS/MS dimension.

#### 2.2.3. Qualitative and Quantitative Data Analysis for Small Molecules

Compound Discoverer and Xcalibur (Thermo, San Jose, CA, USA) were employed for assignment of monoisotopic mass shifts and assignments to specific composition changes using the manufacturer’s recommended settings for the QE LC/MS/MS system with High-energy collisional dissociation (HCD), including 5–10 ppm mass tolerance for extracted ion chromatograms.

#### 2.2.4. Qualitative Data Analysis of Peptides from Digests of Large Molecules

Both Pepfinder (Thermo, San Jose, CA, USA) and Byonic/Byologic (Protein Metrics, San Carlos, CA, USA) were employed for sequence database searching of MS and MS/MS spectra and assignment of monoisotopic mass shifts, and assignments to specific composition changes, in a manner similar to that previously described [2]. Both Pepfinder and Byonic/Byologic settings included the following: two missed cleavages, no static modifications, and the amino acid residue-specific variable modifications discussed in the main text. Several precautions were taken to minimize the omission of low abundance peptides due to inadvertent data reduction during automated or manual data analysis. First, we combined the accurate mass and time-tag strategy of LC/MS-based peak assignments with PepFinder (PepFinder 2014, Thermo Fisher Scientific, 168 Third Avenue Waltham, MA 02451, USA), and Skyline (Skyline v 4.2 MacCoss Lab Software, University of Washington, Department of Genome SciencesFoege, Building S113, 3720 15th Ave NE, Seattle, WA 98195, USA) with the ‘shotgun’ proteomics strategy of LC/MS/MS-based peak assignments with Pepfinder and Byonic/Byologic (Protein Metrics, 20863 Stevens Creek Blvd #450, Cupertino, CA 95014, USA). Moreover, Xcalibur (Xcalibur 3.1, Thermo Fisher Scientific, 168 Third Avenue Waltham, MA 02451, USA), and Skyline assignments were based on a manual inspection of LC/MS, LC/MS/MS, and UV absorption data. Second, we employed relatively less stringent settings for each of these tools in combination than we might have if we had used any one tool alone. Pepfinder and Byonic/Byologic each contained a single amino acid sequence for each of the heavy and light chains of the unconjugated antibody and ADC. Pepfinder used both LC/MS and LC/MS/MS for identification, while Byonic/Byologic used only LC/MS/MS. Pepfinder and Byonic/Byologic were employed with the manufacturer’s recommended settings for the QE LC/MS/MS system with HCD, including 5–10 ppm mass tolerance for extracted ion chromatograms.

#### 2.2.5. Quantitative Data Analysis of Peptides from Digests of Large Molecules

Based on the results of the sequence database search, Skyline was used to detect and quantify the chromatographic peaks in the LC/MS and LC/MS/MS profiles for the unconjugated antibody and the ADC in a manner similar to that previously described [2]. The Skyline settings included mass resolution of 70,000 at *m*/*z* 200, charge states +1 to +6, isotopes 0 to 4 (1st to 5th isotope), number of amino acid residues 0 to 70, and either the MS filter or the MS/MS-based library were used to automatically select peaks in extracted ion chromatograms prior to manual verification. A report was exported for each sample, which defined a peptide feature as a combination of charge state, isotope, and modification, and quantified each peptide feature by the area under the curve. Subtle changes in retention time across samples were sometimes observed due to imperfect chromatographic reproducibility. Therefore, manual verification of qualitative peak assignments with Pepfinder and Byonic/Byologic was performed within Xcalibur (Thermo) and Skyline. This included both a verification of a match between the theoretical and observed isotopic envelopes and a verification of consistent selection of retention times for integration of peaks in extracted ion chromatograms. Problematic peaks were corrected or removed as appropriate. For example, correct integration of chromatographically resolved isobaric species, such as RED-106-modified peptides from the ADC digest, oftentimes required the selection of a relatively wide retention time window in order to capture all important peptide features. Finally, peptide features that were incorrectly selected, as revealed by low (e.g., <0.7) isotopic dot product scores (idotp, a measure of the match to the theoretical isotopic envelope), or MS/MS-based peak assignments more than 5 min from the MS/MS library were removed. The Xcalibur settings included a 5–10 ppm mass tolerance for extracted ion chromatograms and no abundance threshold. The data were exported into a custom report within Skyline for downstream analysis with R. The report included numerous fields for each peptide feature, including the idotp (where the measured peak areas are being compared with the predicted isotope distribution based on the chemical formula for the peptide or molecule in question and the normalized spectral contrast angle or 1-Acos(angle)*2/Pi, where the angle in question is the result of a normal dot-product calculation), isotope- and charge-based peak area, retention time, sequence, and modified peptide sequence.

## 3. Results

**Figure 1:**
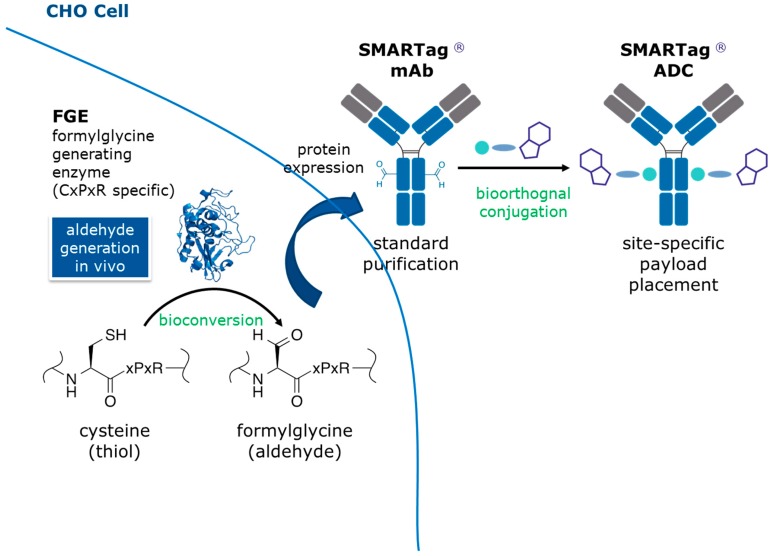
The aldehyde tag technology: A simple and efficient approach for generating site-specific therapeutic bioconjugates. CHO cell, Chinese hamster ovary cell; mAb, monoclonal antibody; SMARTag^®^, Site Specific Modifiable Aldehyde Recombinant Tag; ADC, antibody–drug conjugate.

The aldehyde tag technology is a simple and efficient approach for generating site-specific therapeutic bioconjugates (Figure 1) [10,11,12]. Formylglycine generating enzyme (FGE) is coexpressed with an antibody containing a CysXxxProXxxArg amino acid sequence tag (i.e., the aldehyde tag). FGE catalyzes the cotranslational bioconversion of the thiol group in the Cysteine or free thiol (Cys) residue within the tag sequence to an aldehyde group within a formylglycine (fGly) residue. The antibody is purified using standard techniques (e.g., Protein A affinity chromatography) and is ready for site-specific conjugation to an aldehyde-reactive linker/payload. We favor the Hydrazino-*iso*-Pictet Spengler (HIPS) ligation, which yields a carbon-carbon bond between the chemical linker and the fGly residue within the antibody. Previous LC/MS/MS analyses of ADCs generated using this technology described only the expected modifications of the large or small molecule components [6,13]. Here, we tested a holistic analytic strategy designed to gather information on the linker/payload in isolation and then apply that knowledge to the large molecule conjugate. For this case study, we used an ADC made with our proprietary technology that employs a *C*-terminally aldehyde tagged antibody conjugated to an aldehyde-reactive noncleavable, HIPS-maytansine linker/payload called RED-106 [7].

We began our work by focusing on the small molecule component. Due to its small size, we reasoned that it would be relatively easy to determine the molecular structure of detected analytes, even those representing compounds bearing unknown modifications. We expected no modifications in the RED-106 reference samples, so to identify modifications to RED-106 that occurred as the result of handling or conjugation conditions, we subjected some samples to forced degradation conditions (overnight at ambient temperature in DMA). We also generated two different “surrogate conjugate” samples by conjugating RED-106 to two different aldehyde-containing small molecules (isobutyraldehyde (IBA) or 3-hydroxy-4-methoxybenzaldehyde (HMBA)). Two time points, 1 and 28 h, were monitored for these conjugations.

**Figure 2:**
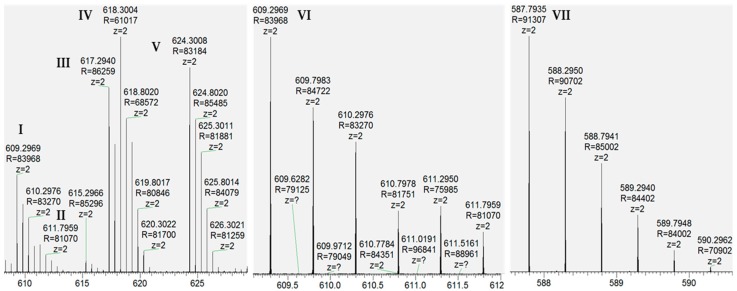
LC/MS/MS revealed modifications of RED-106. Average MS spectra reveal precursor ions as follows: (I) RED-106(−18.008), (II) RED-106(−14.013), (III) RED-106(−2.014), (IV) RED-106, (V) RED-106(+12.001), (VI) RED-106(−18.008, same as I, zoom), (VII) RED-106(−61.015).

**Figure 3:**
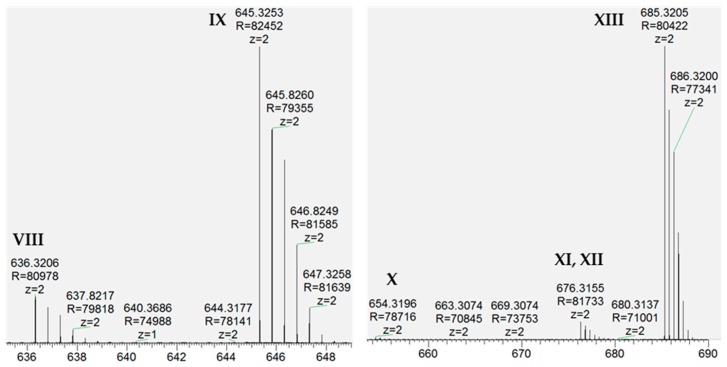
LC/MS/MS revealed modifications of RED-106 in surrogate conjugation reactions. Average MS spectra for the base peaks in panel I reveal precursor ions VIII–XIII, including: (VIII) RED-106(+IBA−18.008), (IX) RED-106(+IBA), (X) RED-106(+HMBA−61.015), (XI) RED-106(+HMBA−18.008), (XII) RED-106(+HMBA−14.013), (XIII) RED-106(+HMBA).

LC/MS/MS of this material revealed previously known and unknown modifications of RED-106 as identified by mass shifts observed relative to the reference mass. We expected a subset of the modifications observed in the degraded RED-106 material to be previously known maytansinoid modifications (e.g., loss of water, detected as −18.008 Da), and another subset to be previously identified HIPS modifications (e.g., formaldehyde addition, detected as +12.001 Da). We confirmed our molecular formula (composition change) assignments for the modifications from the mass shifts observed with LC/MS/MS. Specifically, we detected the following mass shifts in degraded RED-106 and surrogate conjugation samples (in Da): −2.014, +12.001, −14.013, −18.008, and −61.015 (Figure 2 and Table S1). LC/MS/MS revealed that some of these RED-106 modifications (mass shifts) were also observed in the small-molecule conjugates, suggesting that RED-106 species bearing these modifications were conjugatable (Figure 3). By contrast, other RED-106 modifications were not observed in the small-molecule conjugates, suggesting that the RED-106 species bearing these modifications were nonconjugatable.

**Figure 4:**
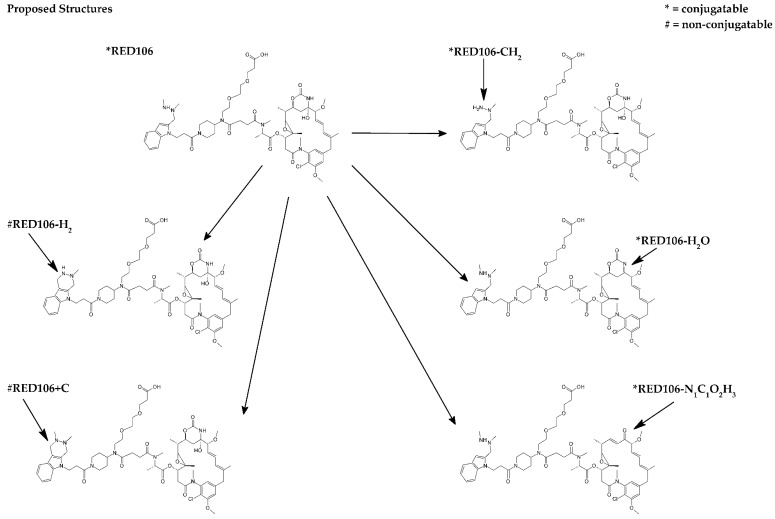
Proposed structures for conjugatable and nonconjugatable modifications of RED-106.

Proposed structures for the modifications are shown in Figure 4. The modifications represented either known maytansine degradants or degradation products of the reactive HIPS indole group. The former class were conjugatable (RED-106-H_2_O, −18.008 Da and RED-106-NCO_2_H_3_, −61.015 Da), with the changes to the maytansine core predicted to reduce the payload’s potency [14]. Regarding the latter class, two modifications were known and nonconjugatable (RED-106-2H, −2.014 Da and RED-106+C, +12.001 Da). We also identified a previously unknown conjugatable HIPS modification (RED-106-CH_2_, −14.013 Da), demonstrating the power of our approach. The proposed structures for the conjugatable and nonconjugatable modifications are supported with evidence from product ion assignments, literature reports [14], and previous studies (data not shown).

We expected no changes in the conjugation-related modifications observed in the small molecule surrogate conjugates as compared to those observed in RED-106-modified peptides from ADC digests. Indeed, LC/MS/MS revealed identical RED-106-related modifications in those two samples (Tables S1 and S2). Moreover, chromatographically resolved stereoisomers arising from the fGly and fGly-HIPS stereocenters were only observed in the RED-106-modified peptides, as expected. As described in the Methods section and shown in Figure S1, correct integration of chromatographically resolved isobaric (same mass) species, such as RED-106-modified peptides and conjugation-related modifications of these peptides, oftentimes required the selection of a relatively wide retention time window to capture all important peptide features. An fGly stereocenter epimerizes readily either as an aldehyde or a hydrozonium intermediate in the bioorthogonal conjugation reaction to yield two stereoisomers in the unconjugated fGly residue. A new stereocenter is formed upon fGly–HIPS conjugation to yield two stereoisomers for the RED-106 conjugated peptide. Therefore, fGly–HIPS conjugation yields four expected isobaric stereoisomers that can be partially or fully chromatographically resolved with LC/MS/MS (see Figure S1), depending on the HIPS linker/payload, the underlying peptide sequence, and the separation conditions.

We observed no changes in the nonconjugation-related modifications detected in peptides from digests of unconjugated antibody as compared to those observed in peptides from digests of the ADC, other than those modifications related to the addition of the linker/payload itself. Akin to our previous work [2], LC/MS/MS revealed numerous nonconjugation-related modifications corresponding to PTMs, such as *N*-linked glycosylation and oxidation in noncomplementarity determining regions, that were shared in both the unconjugated antibody and the ADC samples. However, conjugation-related modification differences were observed when we compared digests of the unconjugated antibody to digests of the ADC (Table S2). LC/MS/MS of peptides from unconjugated antibody digests revealed nearly quantitative (>95%) bioconversion of Cys to fGly within the aldehyde tag sequence with no off-target effects (i.e., no introduction of fGly at other sites). The remaining 5% of the aldehyde tag-modified peptide was comprised of hydrated fGly, glycine (derived from oxidation of fGly), and unconverted Cys (free thiol). LC/MS/MS of the RED-106-modified aldehyde tag-modified peptides from ADC digests revealed nearly quantitative bioorthogonal conjugation (>95%) of fGly with RED-106 with no off-target effects (i.e., no RED-106 ligation at other amino acid residues). These observations were confirmed by using HIC analysis of the ADC, which detected an average DAR of 1.8 (data not shown). Overall, these results reflected site-specific, 95% bioconversion, and 95% bioorthogonal conjugation for each of the two aldehyde-tag-containing heavy chains in this case study (i.e., 1.8 = 0.95 × 0.95 × 2).

## 4. Discussion

The low levels of modifications in reference (“unmodified”) samples are seldom sufficient for developing stability-indicating characterization methods for the extended characterization of ADCs. Therefore, forced degradation of small and large molecule components is often performed for method development purposes alone. This work shows that surrogate small-molecule conjugations in combination with partially degraded linker/payload samples can be essential for understanding and assigning modifications detected in the ADC. Previously known modifications [6] were quickly confirmed, and previously unknown modifications were discovered. While the source(s) of some of the modifications described herein are still under investigation, preliminary studies and previous reports [15,16] suggest that trace levels of formaldehyde from solvents and/or containers are responsible for the +12.000 Da (+C) modification that we observed in the forced degradation RED-106 sample. Significantly, the proposed structures for conjugatable modifications of RED-106 where the modifications represent changes to the maytansine core are expected to be less cytotoxic than RED-106 itself. Evidence for this comes from previous work on the potency of maytansine analogues [17] and structures of maytansine metabolites [14].

Mass spectra from LC/MS/MS data revealed that levels of some of the modifications observed in the forced degradation RED-106 sample were also noted in the conjugation reactions with the small molecule aldehydes IBA and HMBA, suggesting that the modifications were conjugatable. These same modifications were also observed on aldehyde-tag-containing peptides from ADC digests, suggesting that the modifications were reactive with fGly. Other modifications noted in the degraded RED-106 sample were not observed in either the surrogate small-molecule conjugates or the ADC digests, suggesting that RED-106 species bearing these modifications were unreactive with aldehydes and thus were nonconjugatable. We did not observe significant levels of either type of modification until we forced the linker/payload (RED-106) to partially degrade. Likewise, we did not learn whether the observed linker/payload modifications were conjugatable or nonconjugatable modifications until we analyzed the surrogate small-molecule conjugates, where conjugatable modifications were observed as adducts to both small molecule aldehydes tested. By contrast, nonconjugatable modifications were not observed as conjugation products with either of the aldehyde-containing reagents. As additional confirmation of our assignment of conjugatable versus nonconjugatable modifications, we observed identical conjugation-related modifications in the surrogate small-molecule conjugates and the conjugated peptides in ADC digests.

Significantly, nearly quantitative bioconversion and bioorthogonal conjugation were observed at the expected amino acid residues in the unconjugated and conjugated antibody (ADC) samples, respectively, with no off-target effects. This is in stark contrast to cysteine- and lysine-conjugated ADCs, where low levels of unintentional conjugation are often observed at reactive and/or engineered amino acid residues. Thus, bioconversion and bioorthogonal conjugation are highly selective in aldehyde-tagged ADCs conjugated using HIPS chemistry, and the lack of off-target effects is expected to provide improved safety benefits for patients [7].

Our assignments of conjugation-related modifications detected in the surrogate small-molecule conjugates and on specific amino acid residue(s) in peptides from ADC digests, and of nonconjugation-related modifications (e.g., PTMs) found on specific amino acid residue(s) in peptides shared between digests of the unconjugated antibody and ADC were internally consistent. This achievement greatly increased overall confidence in the data set, and was the natural outcome of our hypothesis-driven experimental design. Namely, we applied different experimental and computational bioanalytical chemistry tools in combination, each tailored to discover modifications of small or large molecular components of ADCs. In other words, we showed that differential analysis of monoisotopic mass shifts and assignments to specific composition changes—achieved through a focus on pairwise comparisons of small and large molecular components of therapeutic bioconjugates—enabled the dissection of conjugatable versus nonconjugatable modifications. Our data weave an internally consistent molecular story for our case study on a *C*-terminally-tagged SMARTag^®^ ADC with a proprietary, non-cleavable, aldehyde-reactive HIPS-maytansinoid linker/payload called RED-106.

In conclusion, we hypothesized that LC/MS/MS investigations of an ADC, its molecular components, and surrogate small-molecule conjugates, might be a simple and efficient approach to systematically answer key questions about conjugatable and nonconjugatable modifications for the IND-enabling extended characterization of ADCs. Our hypothesis was based on our previous work, including the importance of replicate characterization studies and appropriate statistical modeling in order to provide reproducible, accurate, and efficient site occupancy estimation and differential analysis [2]. We have observed that comprehensive gas-phase fragmentation is necessary to generate high quality MS/MS spectra for unambiguous assignment of particular modifications. However, in the context of ADCs, this requirement is often an insurmountable challenge for a single laboratory because diverse and advanced expertise, instrumentation, methods, and algorithms have evolved for small versus large molecules, and underlying assumptions might not be correct, particularly for low-level modifications. For example, conjugatable modifications of RED-106 are labile, with isotopes of ^35^Cl and ^37^Cl that are not expected in nonconjugation-related modifications of large molecule unconjugated antibodies, possibly explaining why no conjugation-related modifications of peptides from RED-106 ADC digests were observed when we applied experimental and computational bioanalytical chemistry tools in isolation. Moreover, comprehensive gas-phase fragmentation of labile linker/payload-modified peptides from ADC digests and their unambiguous assignment is problematic (unpublished work). For example, complications arising from isotopic envelopes that differ from averagine—a hypothetical amino acid residue that is often used to model isotopic envelopes in computational tools—have been previously reported [18]. Low-level combinations of modifications (e.g., RED-106-CH_2_-H_2_O), while not discussed herein, further challenge existing capabilities for unambiguous peak assignment. Lastly, isobaric species, such as RED106-modified peptides (Figure S1), must be chromatographically resolved for unambiguous peak assignment. Therefore, chiral separation-based methods merit further investigation. While experimental and computational bioanalytical chemistry tools are rapidly improving for analysis of both the small and large molecule components of ADCs in isolation, we fully anticipate that our strategy of systematic LC/MS/MS investigations for the IND-enabling extended characterization of ADC modifications can be successfully applied to other ADCs and other types of therapeutic bioconjugates.

## Supplementary Materials

The following are available online at http://www.mdpi.com/2073-4468/7/4/40/s1, Table S1: Qualitative LC/MS/MS results for analysis of the degraded RED-106 linker/payload and the surrogate small-molecule conjugates. Table S2: Qualitative LC/MS/MS results for peptides from digests of unconjugated antibody and ADC. Figure S1: Representative chromatograms and isotopic envelopes for RED-106-, RED-106(-CH2)-, RED-106(-N1C1O2H3)- and RED-106(-H2O)-modified SMARTag^®^ peptides, with structure illustrating the position of the fGly and fGly–HIPS stereocenters.

## Abbreviations

ADME	absorption, distribution, metabolism, and excretion
DMA	dimethylacetamide
IBA	isobutyraldehyde
IAA	iodoacetamide
DTT	dithiothreitol
BHT	butylated hydroxytoluene
CAM	carbamidomethylation
MeOx	methoxylamine
Cys	Cysteine or free thiol
ADC	antibody-drug conjugate
fGly	formylglycine
FGE	formylglycine generating enzyme
HIPS	Hydrazino-*iso*-Pictet-Spengler
IND	investigational new drug
LC/MS/MS	liquid chromatography-tandem mass spectrometry
*m/z*	mass-to-charge
mAb	monoclonal antibody
m	monoisotopic mass
PTMs	posttranslational modifications
XIC	extracted ion chromatogram
SMARTag^®^	Site Specific Modifiable Aldehyde Recombinant Tag
HCD	High-energy collisional dissociation
CHO cell	Chinese hamster ovary cell
RED	Redwood Bioscience Inc.
