# Supplementary material for: Systematic LC/MS/MS Investigations for the IND-Enabling Extended Characterization of Antibody–Drug Conjugate Modifications
Source: Antibodies (Basel). 2018 Nov 16;7(4):40. doi: 10.3390/antib7040040 (PMC6698958; doi:10.3390/antib7040040)
Supplement: Supplementary file 1 [file antibodies-07-00040-s001.pdf]

Supplementary information

Table S1. Qualitative LC/MS/MS results for analysis of the degraded RED-106 linker/payload and the surrogate small molecule conjugates.

Small Molecule	Molecular Formula	Theoretical Monoisotopic Mass (m, Da)	Most Abundant Feature Observed			Mass Error (ppm)	Observed Monoisotopic Mass Shift of Modification (Da)	Theoretical Monoisotopic Mass Shift of Modification (Da)	Composition Change of Modification	Observed? (* or #)
			m/z	z	$[m+zH]^{z+}$, where H=1.007825 Da					
RED-106	$C_{62}H_{87}ClN_8O_{16}$	1234.592857	618.3011	2	1236.6022	5.1	0	0	N/A	Y
RED-106àRED-106-H_2O	$C_{62}H_{85}ClN_8O_{15}$	1216.582292	609.2969	2	1218.5938	3.4	-18.008	-18.0157	(-H_2O)	Y (*)
RED-106àRED-106-NCO_2H_3	$C_{61}H_{84}ClN_7O_{14}$	1173.576478	587.7921 and 587.7935	2	1175.584 and 1175.587	6.9 and 4.4	-61.018 and -61.015	-61.0164	(-NCO_2H_3)	Y (*)
RED-106àRED-106-CH_2	$C_{61}H_{85}ClN_8O_{16}$	1220.577207	611.2945	2	1222.589	3.2	-14.013	-14.0157	(-CH_2)	Y (*)
RED-106àRED-106-H_2	$C_{62}H_{85}ClN_8O_{16}$	1232.577207	617.2939	2	1234.5878	4.1	-2.014	-2.0157	(-H_2)	Y (#)
RED-106àRED-106+C	$C_{63}H_{87}ClN_8O_{16}$	1246.592857	624.3011	2	1248.6022	5.1	12	12	(+C)	Y (#)
IBA	C_4H_8O	72.0575	N/A	N/A	N/A	N/A	N/A	N/A	N/A	N/A

IBAàRED-106(+IBA) = RED-106(+54.098)*	$C_{66}H_{93}ClN_8O_{16}$	1288.63907	645.3255	2	1290.651	2.9	0	0	(+$C_{66}H_{93}ClN_8O_{16}$)	Y (*)
IBAàRED-106(+IBA-H_2O)	$C_{66}H_{91}ClN_8O_{15}$	1270.629242	636.3207	2	1272.6414	2.7	-18.01	-18.0157	(+$C_{66}H_{93}ClN_8O_{16}$-H_2O)	Y (*)
IBAàRED-106(+IBA-NCO_2H_3)	$C_{65}H_{90}ClN_7O_{14}$	1227.623429	614.8167	2	1229.6334	4.6	-61.018	-61.0164	(+$C_{66}H_{93}ClN_8O_{16}$-NCO_2H_3)	Y (*)
IBAàRED-106(+IBA-CH_2)	$C_{65}H_{91}ClN_8O_{16}$	1274.624157	638.3177	2	1276.6354	3.5	-14.016	-14.0157	(+$C_{66}H_{93}ClN_8O_{16}$-CH_2)	Y (*)
IBAàRED-106(+IBA-H_2)	$C_{66}H_{91}ClN_8O_{16}$	1286.624157	N/A	N/A	N/A	N/A	N/A	-2.0157	(+$C_{66}H_{93}ClN_8O_{16}$-H_2)	N (#)
IBAàRED-106(+IBA+C)	$C_{67}H_{93}ClN_8O_{16}$	1300.639807	N/A	N/A	N/A	N/A	N/A	12	(+$C_{66}H_{93}ClN_8O_{16}$+C)	N (#)
HMBA	$C_8H_8O_3$	152.047344	N/A	N/A	N/A	N/A	N/A	N/A	N/A	N/A
HMBAàRED-106(+HMBA) = RED-106(+134.037)*	$C_{70}H_{93}ClN_8O_{18}$	1368.62936	685.3204	2	1370.6408	3.1	0	0	(+$C_{70}H_{93}ClN_8O_{18}$)	Y (*)
HMBAàRED-106(+HMBA-H_2O)	$C_{70}H_{91}ClN_8O_{17}$	1350.619072	676.3154	2	1352.6308	2.9	-18.01	-18.0157	(+$C_{70}H_{93}ClN_8O_{18}$-H_2O)	Y (*)
HMBAàRED-106(+HMBA-NCO_2H_3)	$C_{69}H_{90}ClN_7O_{16}$	1307.613258	654.3196	2	1308.62	755.4 (low S/N)	-62.001	-61.0164	(+$C_{70}H_{93}ClN_8O_{18}$-NCO_2H_3)	Y (*)
HMBAàRED-106(+HMBA-CH_2)	$C_{69}H_{91}ClN_8O_{18}$	1354.613986	678.3175	2	1356.635	-4	-14.006	-14.0157	(+$C_{70}H_{93}ClN_8O_{18}$-CH_2)	Y (*)

**HMBAàRED-106(+HMBA-H_2)**	**$C_{70}H_{91}ClN_8O_{18}$**	**1366.613986**	**N/A**	**N/A**	**N/A**	**N/A**	**N/A**	**-2.0157**	**(+$C_{70}H_{93}ClN_8O_{18}$-H_2)**	**N (#)**
**HMBAàRED-106(+HMBA+C)**	**$C_{71}H_{93}ClN_8O_{18}$**	**1380.629636**	**N/A**	**N/A**	**N/A**	**N/A**	**N/A**	**12**	**(+$C_{70}H_{93}ClN_8O_{18}$+C)**	**N (#)**

Table S2. Qualitative LC/MS/MS results for peptides from digests of unconjugated antibody and ADC.

Aldehyde tag Peptide from Large Molecule	Molecular Formula	Theoretical Monoisotopic Mass (m, Da)	Most Abundant Feature Observed			Mass Error (ppm)	Observed Monoisotopic Mass Shift of Modification (Da)	Theoretical Monoisotopic Mass Shift of Modification (Da)	Composition Change of Modification	Observed in mAb?	Observed in ADC? (* or #)
			m/z	z	$[m+zH]^{z+}$, where H=1.007825 Da						
SLSLSPGSLCTPSR	$C_{58}H_{101}N_{17}O_{21}S_1$	1403.707864	702.861	2	1405.7224	0.8	0	0	N/A	Y	Y
CysàfGly (bioconversion)	$C_{58}H_{99}N_{17}O_{22}$	1385.715058	693.865	2	1387.7296	0.8	17.993	-17.993	$(+O-H_2S)$	Y	Y
CysàfGlyàMeOx (analytical methoxylamine modification for quantification of bioconversion/bioorthogonal conjugation)	$C_{59}H_{102}N_{18}O_{22}$	1414.741607	708.378	2	1416.7562	0.7	-11.034	11.034	(+HCNO-S)	Y	Y
CysàfGlyàGly	$C_{57}H_{99}N_{17}O_{21}$	1357.720144	679.867	2	1359.7346	0.9	45.9878	-45.988	$(-H_2CS)$	Y	Y
CysàfGlyàhydrated fGly	$C_{58}H_{101}N_{17}O_{23}$	1403.725623	702.87	2	1405.7402	0.8	1405	0.0178	$(+H_2O_2-H_2S)$	Y	Y
CysàCys (analytical carbamidomethyl modification for	$C_{60}H_{104}N_{18}O_{22}S_1$	1460.729328	731.372	2	1462.7438	0.8	-57.0214	57.022	$(+H_3C_2NO)$	Y	Y

**quantification of bioconversion/bioorthogonal conjugation)**											
**Glutathionylation on Cys**	**$C_{68}H_{116}N_{20}O_{27}S_2$**	**1708.77602**	**855.395**	**2**	**1710.7906**	**0.6**	**-305.0682**	**305.068**	**(+$H_{15}C_{10}N_3O_6S$)**	**Y**	**Y**
**CysàfGlyàRED-106 (bioorthogonal conjugation)**	**$C_{120}H_{184}N_{25}O_{35}S_1Cl_1$**	**2602.279592**	**868.434**	**3**	**2605.3014**	**0.6**	**-1199.579**	**1198.57127**	**(+$C_{62}H_{83}ClN_8O_{14}$)**	**N/A**	**Y (*)**
**CysàfGlyàRED-106-H_2O**	**$C_{120}H_{182}N_{25}O_{34}S_1Cl_1$**	**2584.269027**	**862.43**	**3**	**2587.2909**	**0.6**	**-1181.5685**	**1180.5556**	**(+$C_{62}H_{83}ClN_8O_{14}$-H_2O)**	**N/A**	**Y (*)**
**CysàfGlyàRED-106-NCO_2H_3**	**$C_{119}H_{181}N_{24}O_{33}S_1Cl_1$**	**2541.263214**	**848.095**	**3**	**2544.285**	**0.7**	**-1138.5626**	**1137.5549**	**(+$C_{62}H_{83}ClN_8O_{14}$-NCO_2H_3)**	**N/A**	**Y (*)**
**CysàfGlyàRED-106-CH_2**	**$C_{119}H_{182}N_{25}O_{35}S_1Cl_1$**	**2588.263942**	**863.762**	**3**	**2591.2857**	**0.7**	**-1185.5633**	**1184.5556**	**(+$C_{62}H_{83}ClN_8O_{14}$-CH_2)**	**N/A**	**Y (*)**
**CysàfGlyàRED-106-H_2**	**$C_{120}H_{184}N_{25}O_{36}S_1Cl_1$**	**2618.274507**	**N/A**	**N/A**	**N/A**	**N/A**	**N/A**	**1196.5556**	**(+$C_{62}H_{83}ClN_8O_{14}$-H_2)**	**N/A**	**N (#)**
**CysàfGlyàRED-106+C**	**$C_{121}H_{186}N_{25}O_{36}S_1Cl_1$**	**2632.290157**	**N/A**	**N/A**	**N/A**	**N/A**	**N/A**	**1210.5713**	**(+$C_{62}H_{83}ClN_8O_{14}$+C)**	**N/A**	**N (#)**

* Conjugatable modifications; # nonconjugatable modifications, Y – yes, N – no, N/A – not applicable.

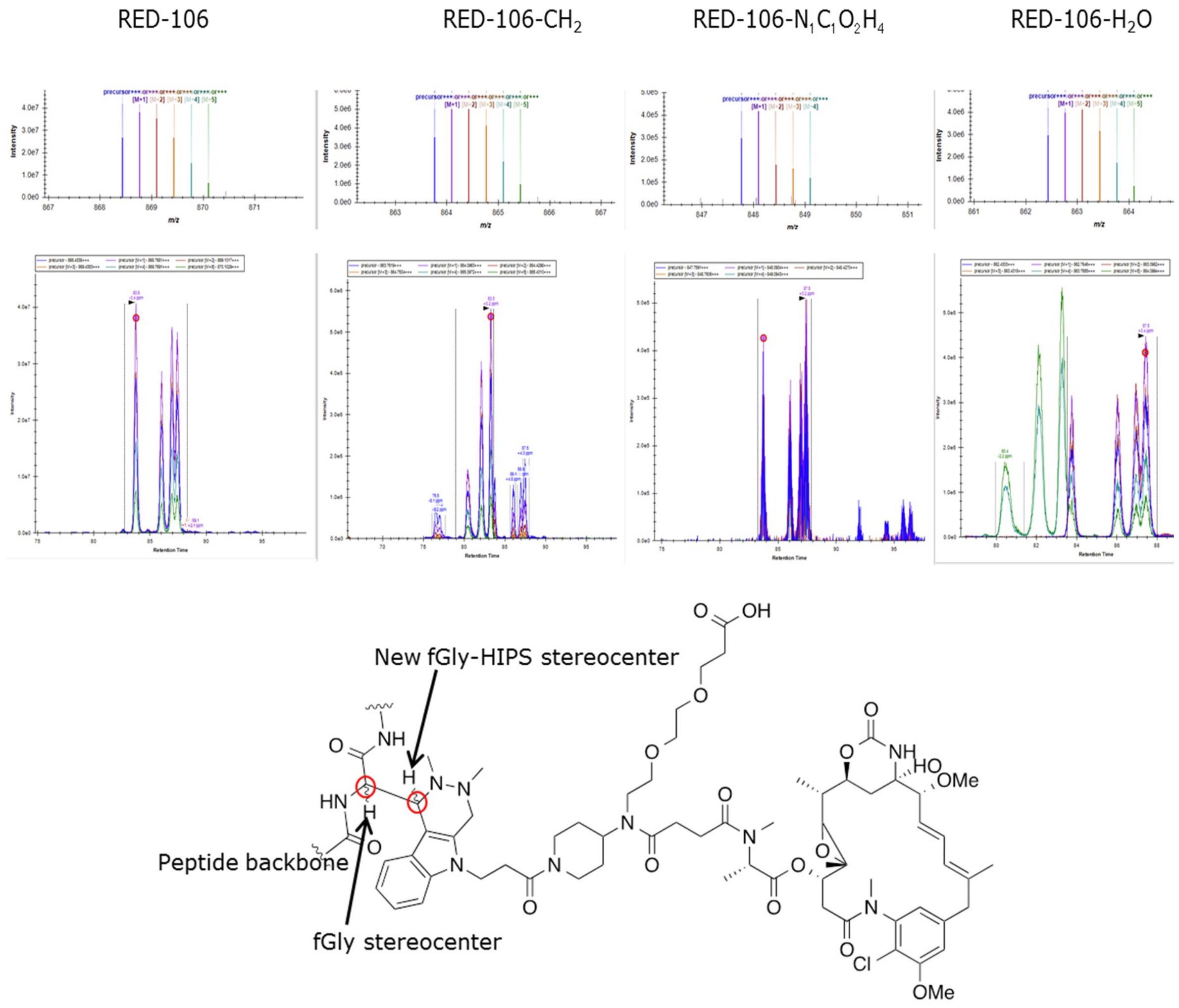

Figure S1. Representative chromatograms and isotopic envelopes for RED-106-, RED-106(-CH2)-, RED-106(-N1C1O2H3)- and RED-106(-H2O)-modified SMARTag® peptides.

LC/MS/MS revealed identical conjugatable modifications in the surrogate conjugates and in aldehyde tag containing peptides from digests of the ADC, including full chromatographic resolution for all four expected isobaric (same mass) stereoisomers arising from the 2 fGly and 2 fGly-HIPS stereocenters in (D) for each of the following (from left to right): RED-106-, RED-106(-CH2)-, RED-106(-$N_1C_1O_2H_3$)- andRED-106(-H_2O)-modified SMARTag® peptides. Strong evidence for the assignment of each chromatographic peak is provided by the consistent isotopic envelopes shown above each extracted ion chromatogram. The structure shown illustrates the position of the fGly and fGly-HIPS stereocenters relative to the peptide backbone in RED-106-modified SMARTag® peptides and conjugation-related modifications of these peptides.
